# Supplementary material for: Regulation of pulmonary surfactant by the adhesion GPCR GPR116/ADGRF5 requires a tethered agonist-mediated activation mechanism
Source: eLife. 2022 Sep 8;11:e69061. doi: 10.7554/eLife.69061 (PMC9489211; doi:10.7554/eLife.69061)
Supplement: Figure 1—source data 1. [file elife-69061-fig1-data1.pptx]

## Slide 1
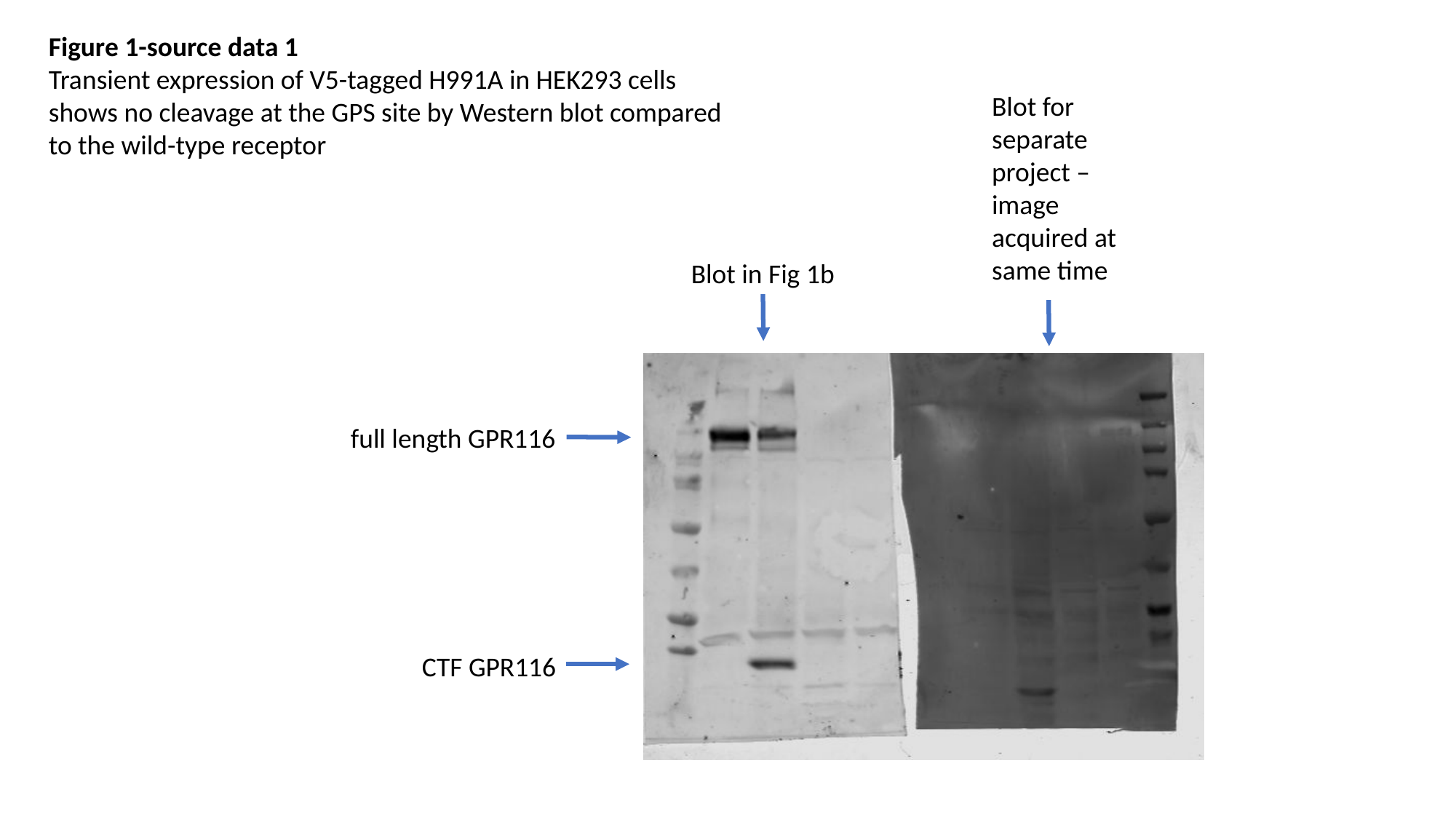

Figure 1-source data 1
Transient expression of V5-tagged H991A in HEK293 cells shows no cleavage at the GPS site by Western blot compared to the wild-type receptor
Blot for separate project –
image acquired at same time
Blot in Fig 1b
full length GPR116
CTF GPR116
