# Supplementary figures and images for: Regulation of pulmonary surfactant by the adhesion GPCR GPR116/ADGRF5 requires a tethered agonist-mediated activation mechanism
Source: eLife. 2022 Sep 8;11:e69061. doi: 10.7554/eLife.69061 (PMC9489211; doi:10.7554/eLife.69061)

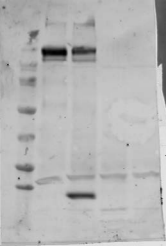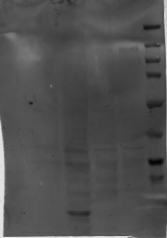

Supplement: Figure 1—source data 2. [file elife-69061-fig1-data2.pdf]

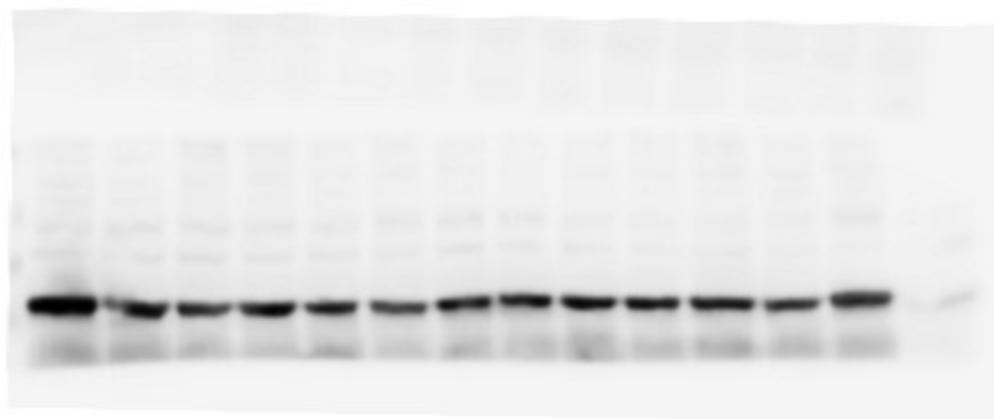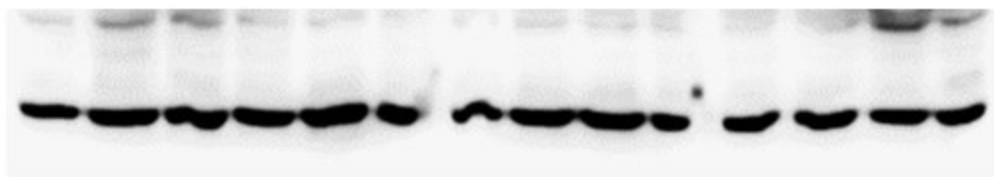

Supplement: Figure 3—source data 1. [file elife-69061-fig3-data1.pdf]

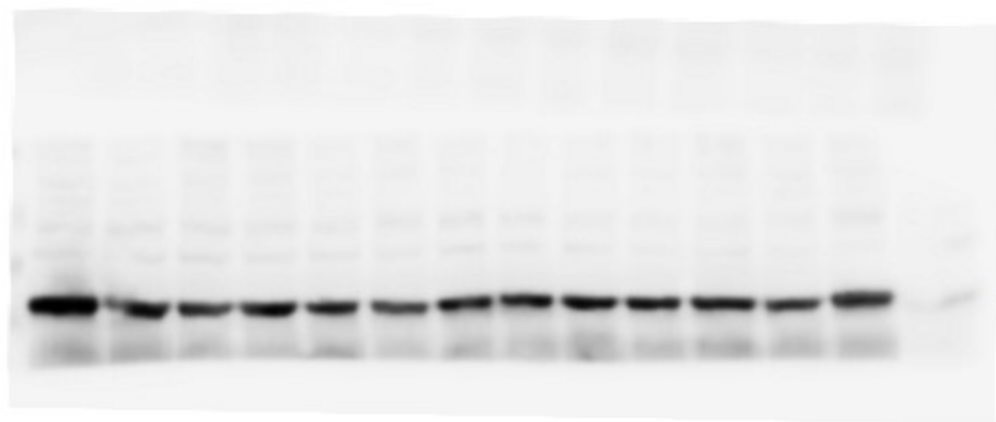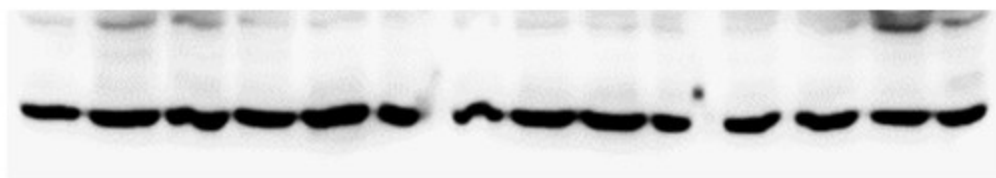

Supplement: Figure 3—source data 2. [file elife-69061-fig3-data2.pdf]

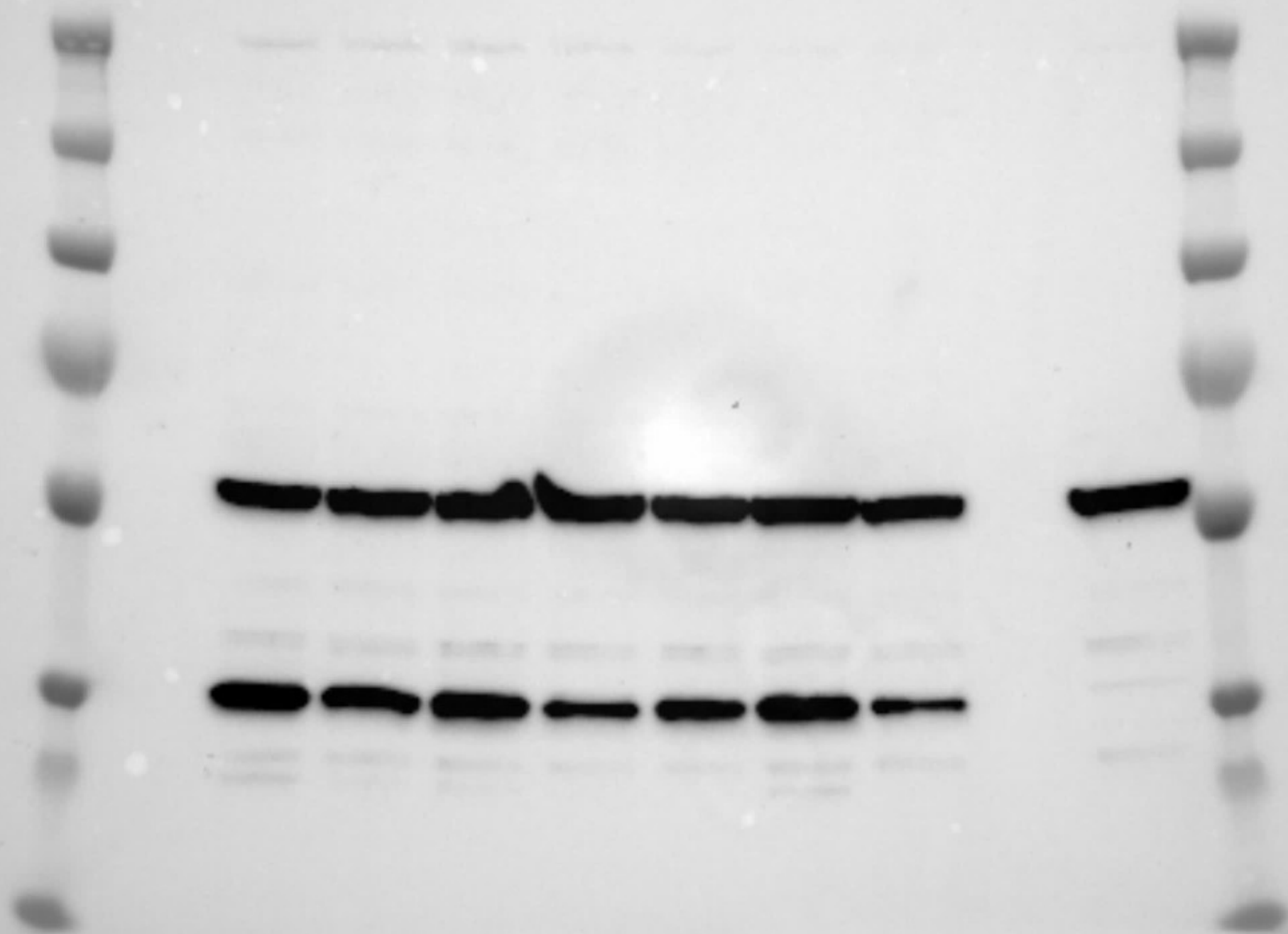

Supplement: Figure 4—source data 1. [file elife-69061-fig4-data1.pdf]

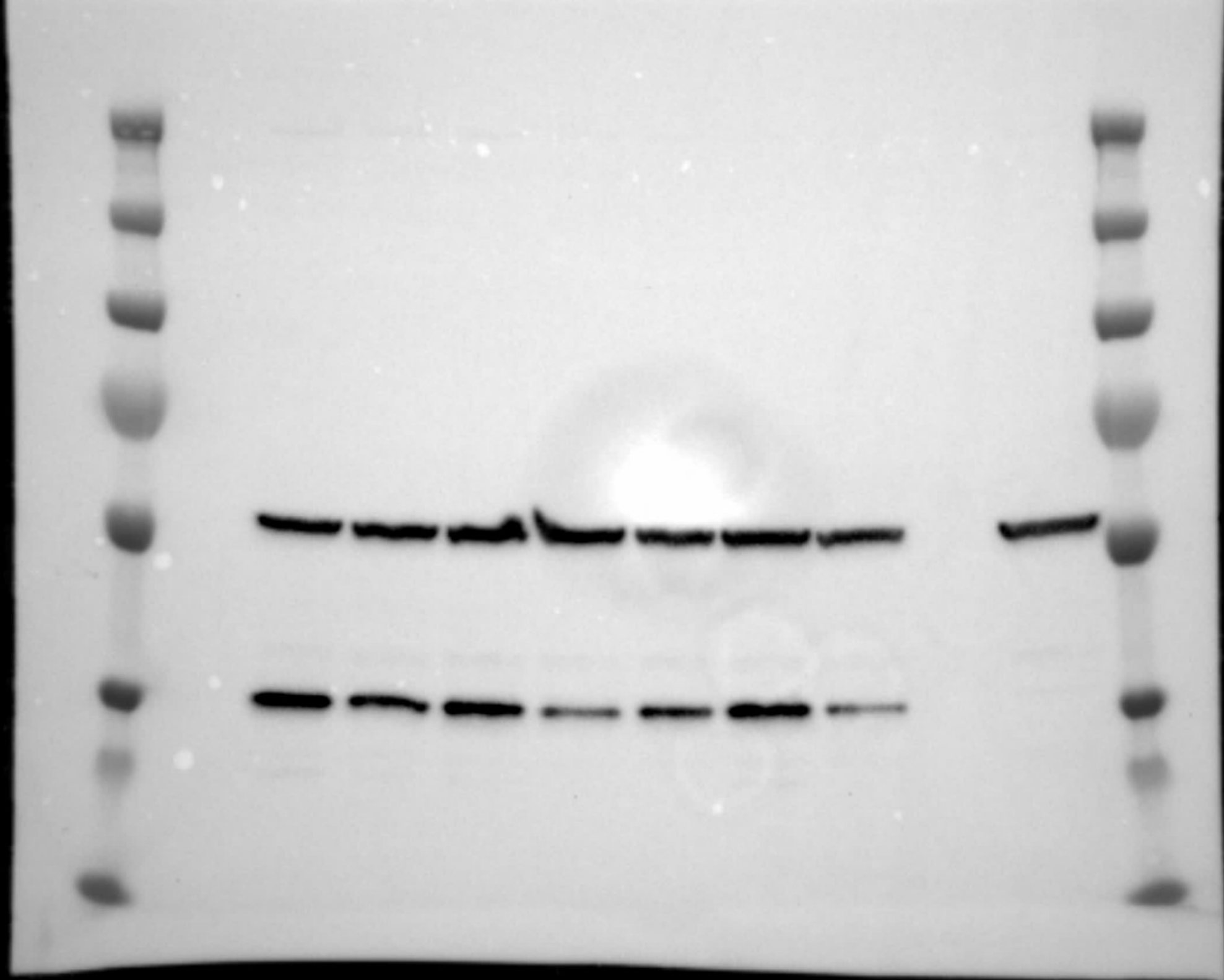

Supplement: Figure 4—source data 2. [file elife-69061-fig4-data2.pdf]

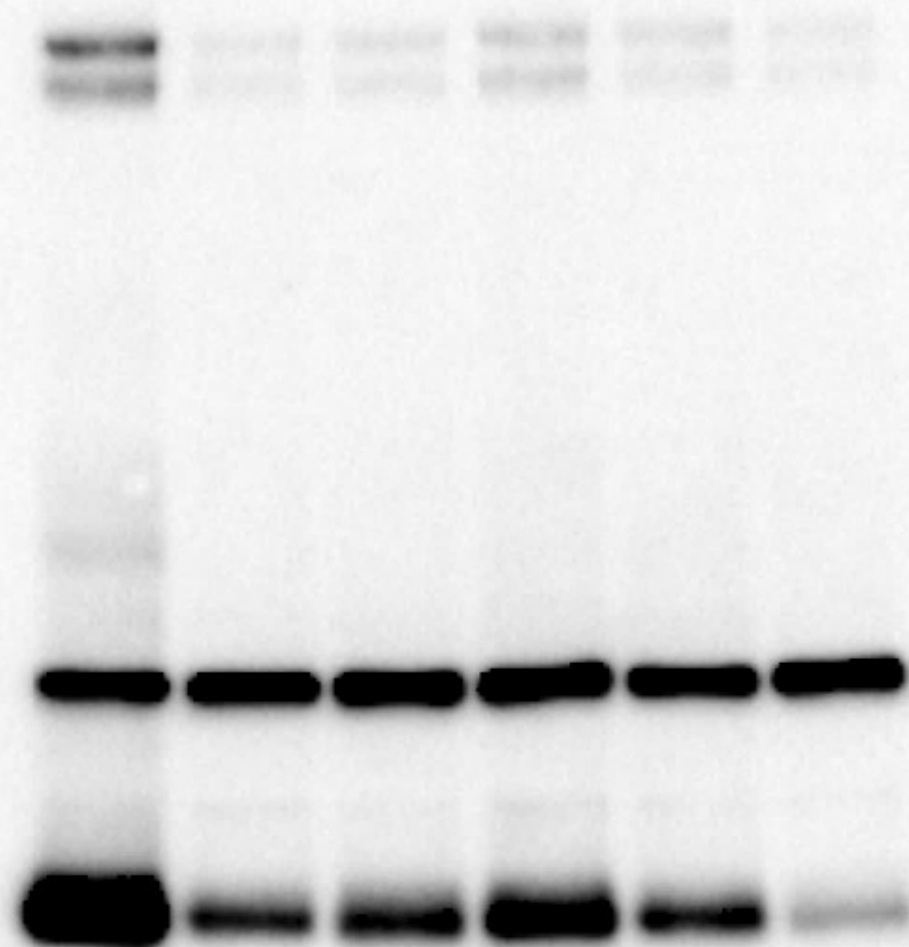

Supplement: Figure 4—source data 5. [file elife-69061-fig4-data5.pdf]

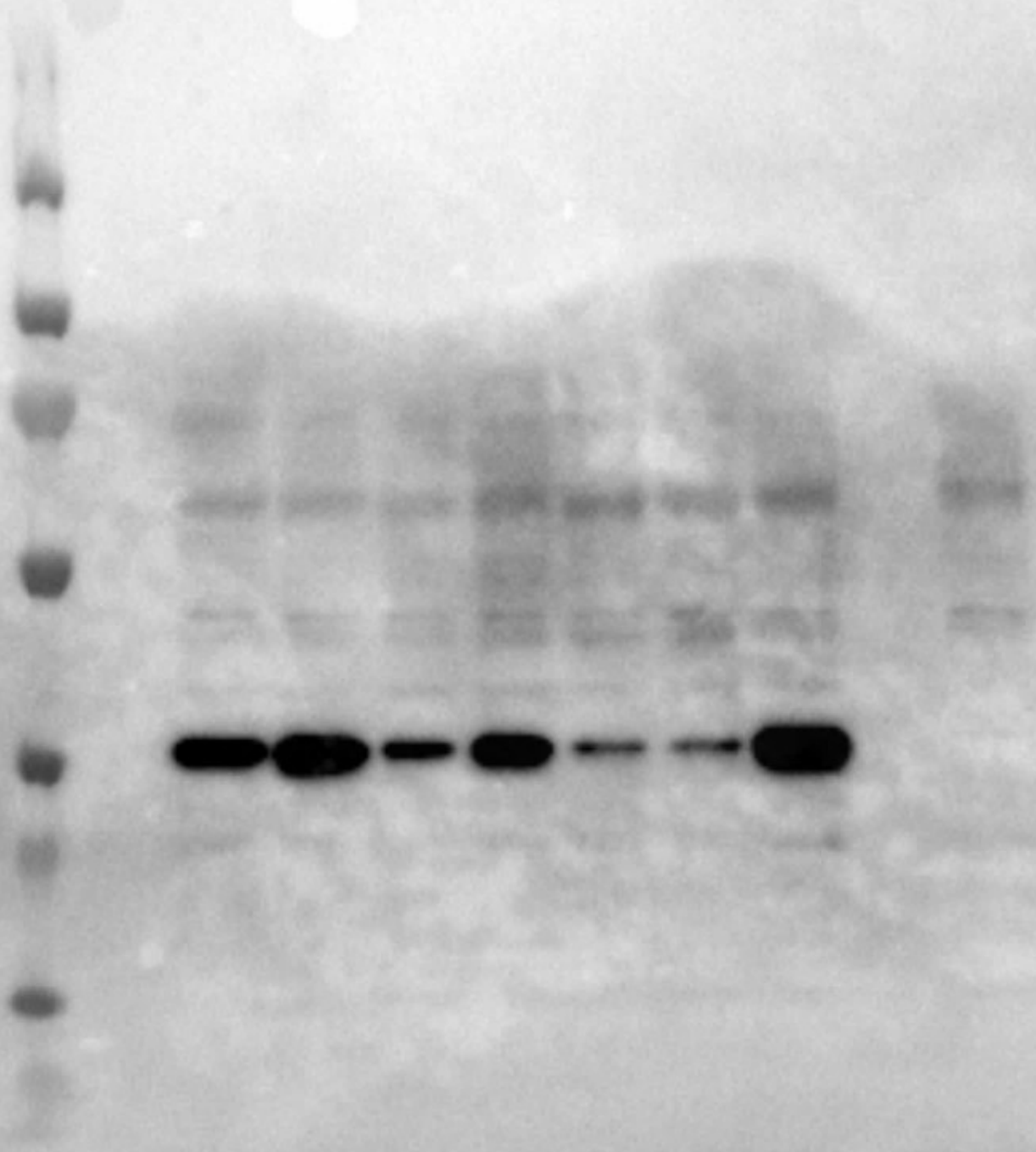

Supplement: Figure 4—source data 7. [file elife-69061-fig4-data7.pdf]

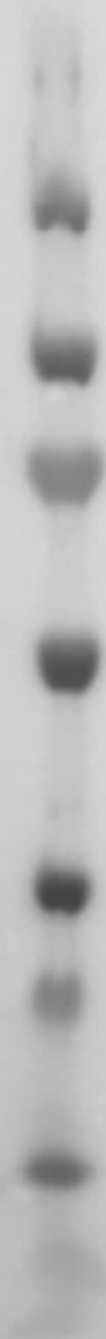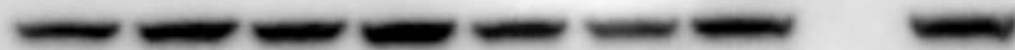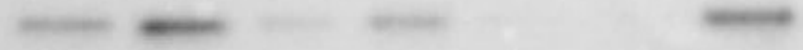

Supplement: Figure 4—source data 8. [file elife-69061-fig4-data8.pdf]

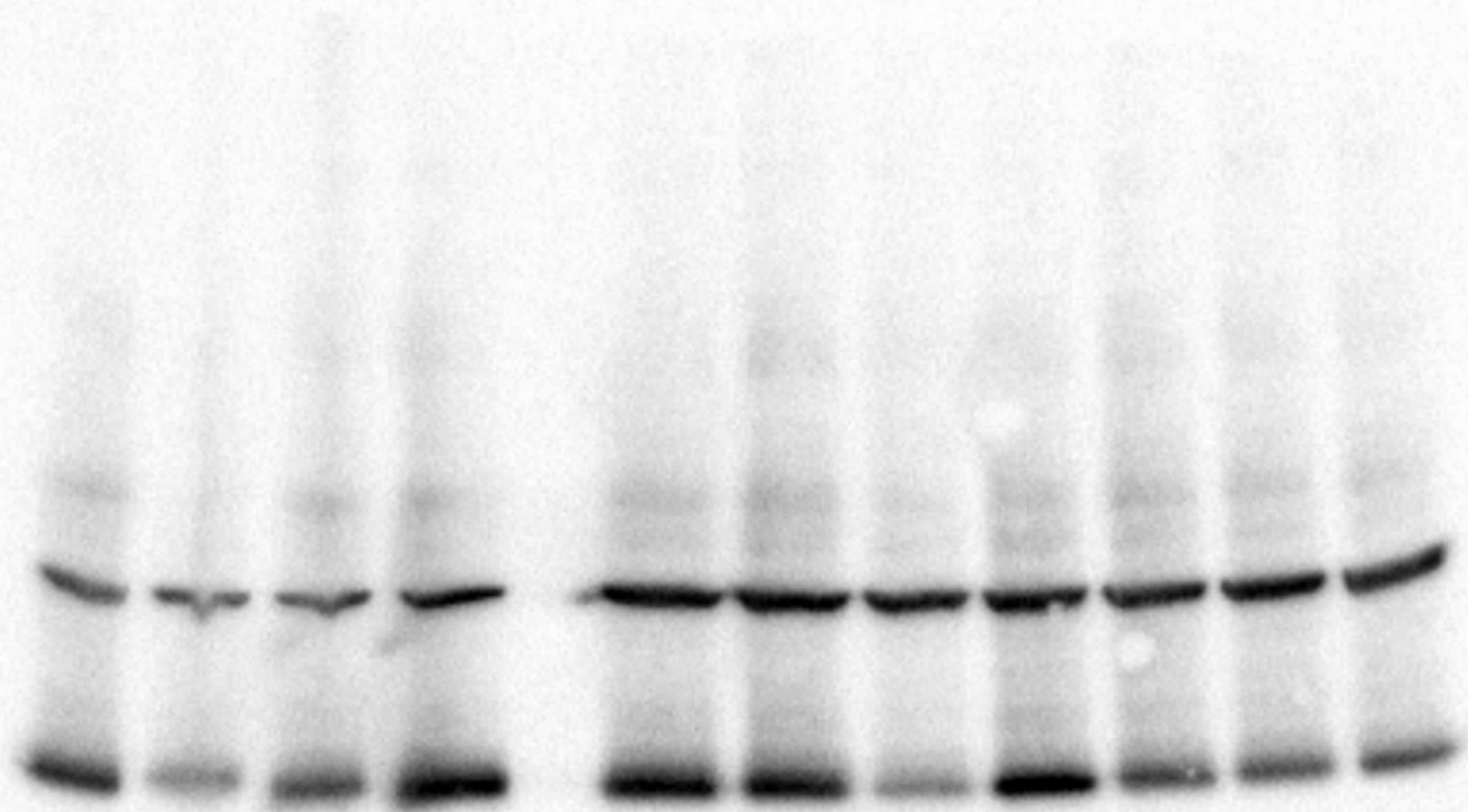

100kDa 100kDa 100kDa 100kDa 100kDa 100kDa 100kDa 100kDa 100kDa 100kDa

Supplement: Figure 5—source data 1. [file elife-69061-fig5-data1.pdf]

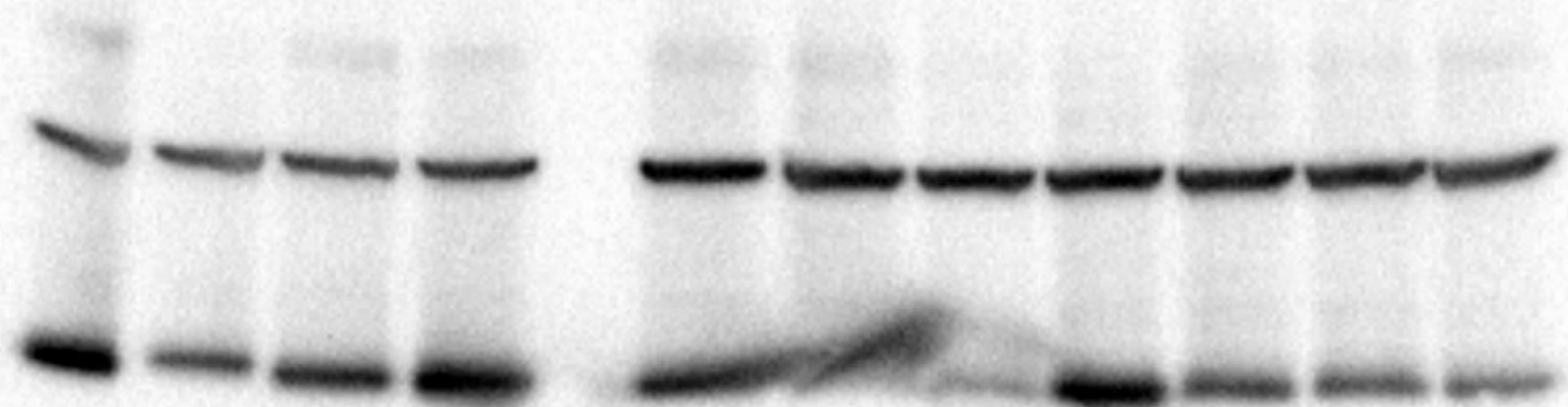

Supplement: Figure 6—source data 1. [file elife-69061-fig6-data1.pdf]
