# Supplementary material for: Regulation of pulmonary surfactant by the adhesion GPCR GPR116/ADGRF5 requires a tethered agonist-mediated activation mechanism
Source: eLife. 2022 Sep 8;11:e69061. doi: 10.7554/eLife.69061 (PMC9489211; doi:10.7554/eLife.69061)
Supplement: Figure 3—source data 3. [file elife-69061-fig3-data3.docx]

**Figure 3-source data 3**

Activation of full-length mGPR116 in stable expressing cells (HEK293 clone 3C) with exogenous GAP10 peptides that were sequentially mutated to alanine at each position. Receptor activation was measured via calcium transient assays, in n=2-3 independent experiments. **P<0.01 ***P<0.001 **** P<0.0001.

|  |  |  | **EC50** | **(uM)** |  | **mean** | **SD** | statistical |
| --- | --- | --- | --- | --- | --- | --- | --- | --- |
|  | **Peptide name** | **Peptide sequence** | n=1 | n=2 | n=3 |  |  | significance |
|  | GAP14 | TSFSILMSPDSPDP | 29.9 | 26.3 | 46.4 | 34.2 | 10.7 | ** |
|  | GAP14-Nle | TSFSILNleSPDSPDP | 30.2 | 26.3 | 23.9 | 26.8 | 3.2 | ns |
|  | GAP10 | TSFSILMSPD | 16.0 | 8.6 | 6.9 | 10.5 | 4.8 |  |
|  | GAP10-Nle | TSFSILNleSPD | 11.3 | 0.6 | 4.1 | 5.3 | 5.5 | ns |
|  | GAP7 | TSFSILNle | >100 | >100 | >100 | >100 |  | **** |
| **Corresponding mCTF mutant** | |  |  |  |  |  |  |  |
| T993A | GAP10-POS1, T -> A | ASFSILNleSPD | 20.2 | 12.0 | 15.5 | 15.9 | 4.1 | ns |
| S994A | GAP10-POS2, S -> A | TAFSILNleSPD | 9.2 | 8.3 | 9.9 | 9.1 | 0.8 | ns |
| F995A | GAP10-POS3, F -> A | TSASILNleSPD | >100 | >100 | >100 | >100 |  | **** |
| S996A | GAP10-POS4, S -> A | TSFAILNleSPD | 18.0 | 19.1 |  | 18.5 | 0.8 | ns |
| I997A | GAP10-POS5, I -> A | TSFSALNleSPD | 50.9 | 29.4 | 45.4 | 41.9 | 11.1 | *** |
| L998A | GAP10-POS6, L -> A | TSFSIANleSPD | >100 | >100 | >100 | >100 |  | **** |
| M999A | GAP10-POS7, M -> A | TSFSILASPD | >100 | >100 | >100 | >100 |  | **** |
| S1000A | GAP10-POS8, S -> A | TSFSILNleAPD | 32.6 | 24.8 | 55.3 | 37.6 | 15.8 | ** |
| P1001A | GAP10-POS9, P -> A | TSFSILNleSAD | 4.3 | 3.4 | 30.6 | 12.7 | 15.4 | ns |
| D1002A | GAP10-POS10, D -> A | TSFSILNleSPA | 11.2 | 12.1 |  | 11.7 | 0.6 | ns |
