# Supplementary material for: Regulation of pulmonary surfactant by the adhesion GPCR GPR116/ADGRF5 requires a tethered agonist-mediated activation mechanism
Source: eLife. 2022 Sep 8;11:e69061. doi: 10.7554/eLife.69061 (PMC9489211; doi:10.7554/eLife.69061)
Supplement: Figure 4—source data 3. [file elife-69061-fig4-data3.pptx]

## Slide 1
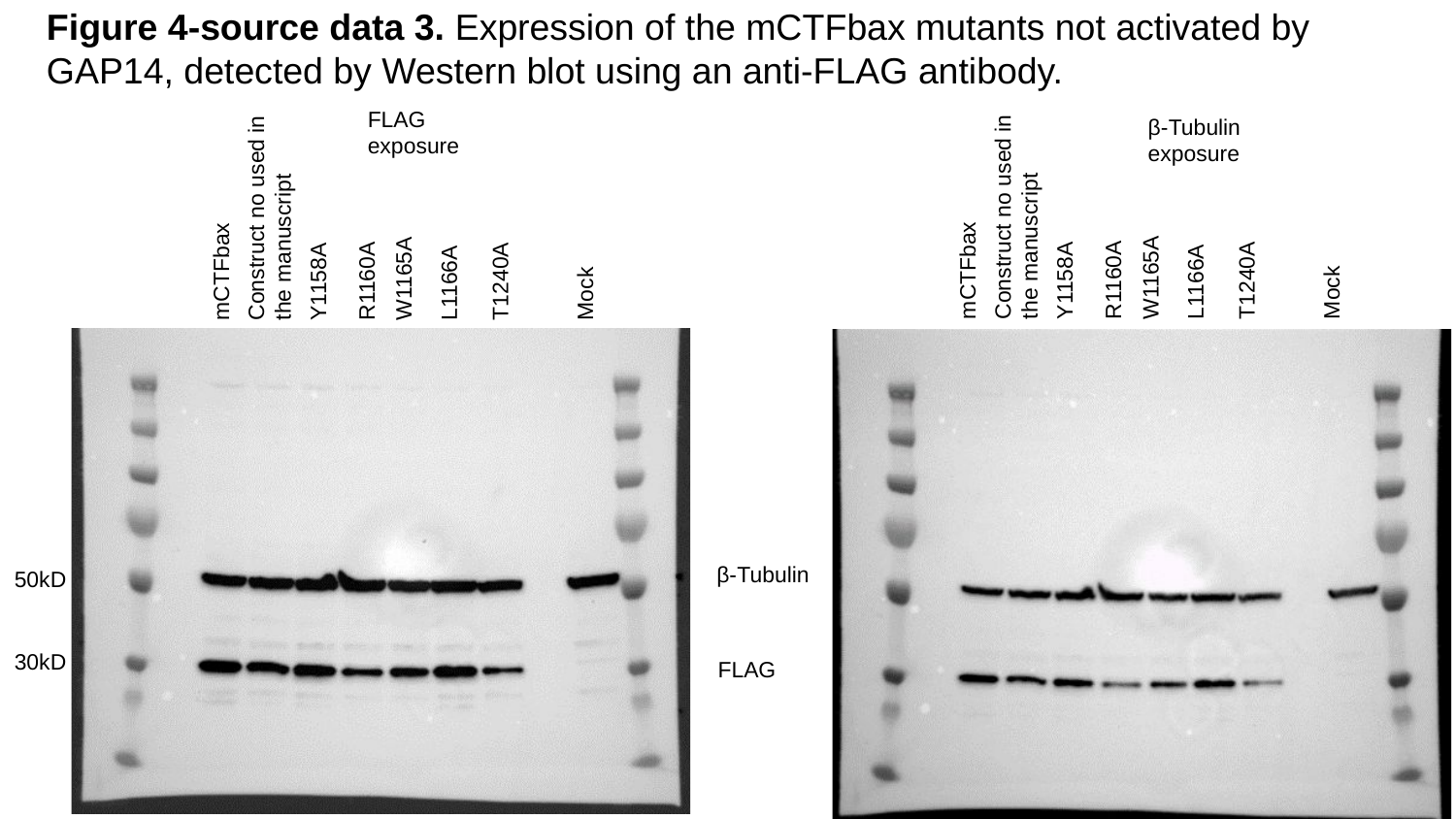

Figure 4-source data 3. Expression of the mCTFbax mutants not activated by GAP14, detected by Western blot using an anti-FLAG antibody.
Construct no used in the manuscript
mCTFbax
Y1158A
R1160A
W1165A
L1166A
T1240A
Mock
β-Tubulin
50kD
30kD
FLAG
FLAG exposure
β-Tubulin exposure
Construct no used in the manuscript
mCTFbax
Y1158A
R1160A
W1165A
L1166A
T1240A
Mock
