# Supplementary material for: Regulation of pulmonary surfactant by the adhesion GPCR GPR116/ADGRF5 requires a tethered agonist-mediated activation mechanism
Source: eLife. 2022 Sep 8;11:e69061. doi: 10.7554/eLife.69061 (PMC9489211; doi:10.7554/eLife.69061)
Supplement: Figure 4—source data 4. [file elife-69061-fig4-data4.docx]

**Figure 4-source data 4. Alignment of the human and mouse GPR116 CTF sequences.**

Residues that are not conserved between the two species are highlighted in red and bold.

|  | **tethered peptide (993 - 1008)** |  |  |  |
| --- | --- | --- | --- | --- |
| **hGPR116 CTF** | TSFSILMSPDSPDP**S**S |  |  |  |
| **mGPR116 CTF** | TSFSILMSPDSPDP**G**S |  |  |  |
|  |  |  |  |  |
|  |  |  |  |  |
|  | **TM1 (1009 - 1038)** | **ICL1 (1039 - 1054)** | **TM2 (1055 - 1080)** | **ECL1 (1081 - 1087)** |
| **hGPR116 CTF** | LL**G**ILLDIISY**V**G**V**GFSI**L**SLAACLVVEA**V** | VWKSVTKNRTSYMRH**T** | CIVNIA**AS**LL**V**A**NT**WFIV**VA**AI**QDN**R | Y**I**L**CK**TA |
| **mGPR116 CTF** | LL**K**ILLDIISY**I**G**L**GFSI**V**SLAACLVVEA**M** | VWKSVTKNRTSYMRH**I** | CIVNIA**FC**LL**I**A**DI**WFIV**AG**AI**HDG**R | Y**P**L**N**ETA |
|  |  |  |  |  |
|  |  |  |  |  |
|  | **TM3 (1088 - 1113)** | **ICL2 (1114 - 1130)** | **TM4 (1131 - 1152)** | **ECL2 (1153 - 1174)** |
| **hGPR116 CTF** | CVAATFFIHFFYLSVFFWMLTLGLML | FYRL**V**FILH**ET**S**R**STQK | AIAF**C**LGYGCPL**A**IS**V**IT**L**G**A**T | QP**R**EVY**T**RKN**V**CWLNWEDT**K**AL |
| **mGPR116 CTF** | CVAATFFIHFFYLSVFFWMLTLGLML | FYRL**I**FILH**DA**S**K**STQK | AIAF**S**LGYGCPL**I**IS**S**IT**V**G**V**T | QP**Q**EVY**M**RKN**A**CWLNWEDT**R**AL |
|  |  |  |  |  |
|  |  |  |  |  |
|  | **TM5 (1175 - 1200)** | **ILC3 (1201 - 1209)** | **TM6 (1210 - 1239)** | **ECL3 (1240 - 1245)** |
| **hGPR116 CTF** | LAFAIPALIIVVVN**IT**IT**I**VVITKIL | RPSIGDKP**C** | KQEKSSLFQISKSIGVLTPLLGLTWGFGL**T** | TV**FPGT** |
| **mGPR116 CTF** | LAFAIPALIIVVVN**VS**IT**V**VVITKIL | RPSIGDKP**G** | KQEKSSLFQISKSIGVLTPLLGLTWGFGL**A** | TV**IQGS** |
|  |  |  |  |  |
|  |  |  |  |  |
|  | **TM7 (1246 - 1271)** | **Ct tail (1272 - 1348)** | |  |
| **hGPR116 CTF** | N**L**VFHIIF**AI**LN**V**FQGLFILLFGCLW | D**L**KVQEALL**N**KFSLSRWSSQHSKSTS**L**GSSTPVFSMSSPISRRFNNLFGKTGTYNVSTPE**A**TSSSLENSSSA**S**SLLN | | |
| **mGPR116 CTF** | N**A**VFHIIF**TL**LN**A**FQGLFILLFGCLW | D**Q**KVQEALL**H**KFSLSRWSSQHSKSTS**I**GSSTPVFSMSSPISRRFNNLFGKTGTYNVSTPE**T**TSSSLENSSSA**Y**SLLN | | |
