# Supplementary material for: Regulation of pulmonary surfactant by the adhesion GPCR GPR116/ADGRF5 requires a tethered agonist-mediated activation mechanism
Source: eLife. 2022 Sep 8;11:e69061. doi: 10.7554/eLife.69061 (PMC9489211; doi:10.7554/eLife.69061)
Supplement: Figure 4—source data 6. [file elife-69061-fig4-data6.pptx]

## Slide 1
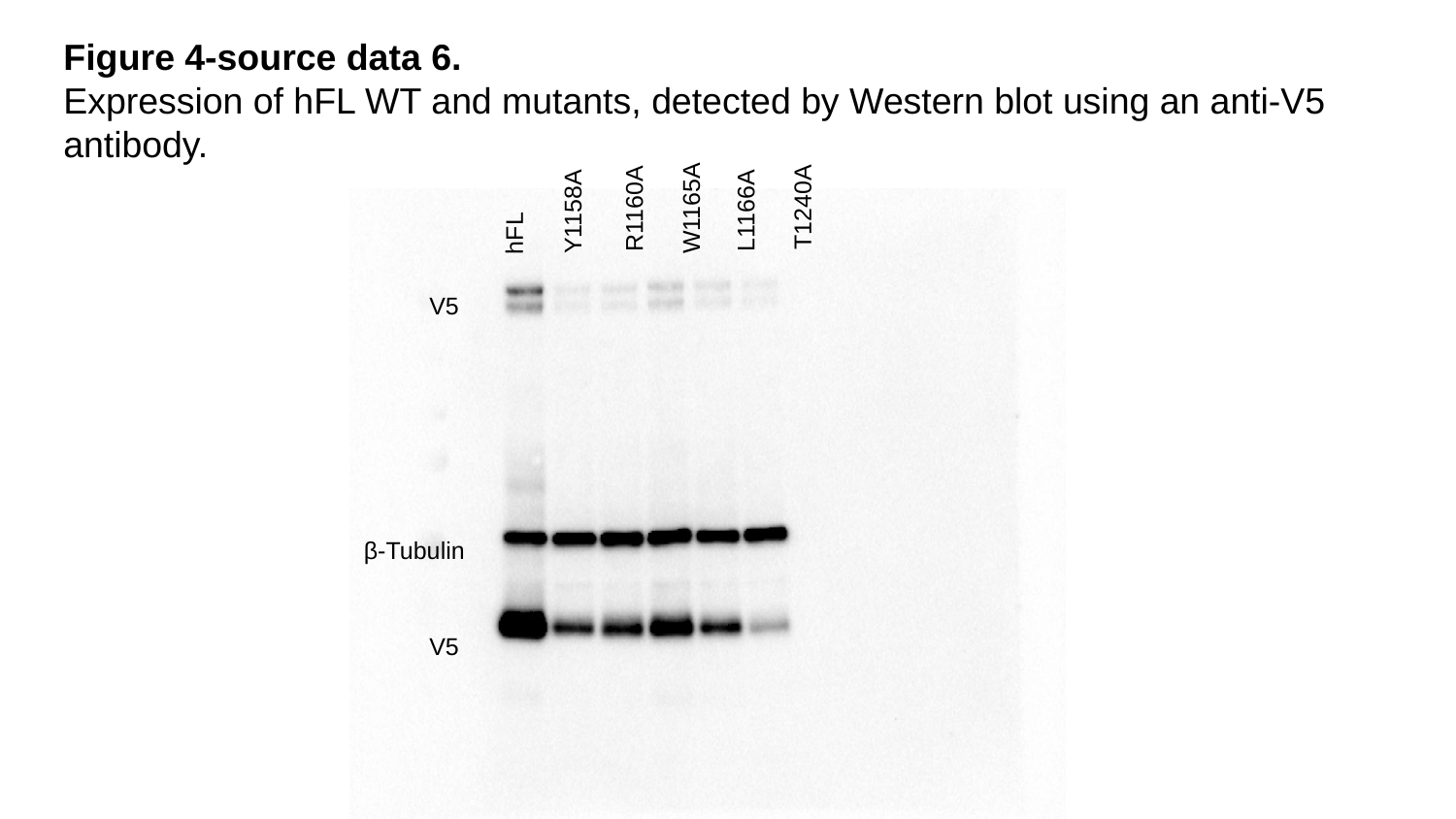

Figure 4-source data 6.
Expression of hFL WT and mutants, detected by Western blot using an anti-V5 antibody.
T1240A
W1165A
L1166A
R1160A
Y1158A
hFL
V5
β-Tubulin
V5
