# Supplementary material for: Regulation of pulmonary surfactant by the adhesion GPCR GPR116/ADGRF5 requires a tethered agonist-mediated activation mechanism
Source: eLife. 2022 Sep 8;11:e69061. doi: 10.7554/eLife.69061 (PMC9489211; doi:10.7554/eLife.69061)
Supplement: Figure 4—source data 9. [file elife-69061-fig4-data9.pptx]

## Slide 1
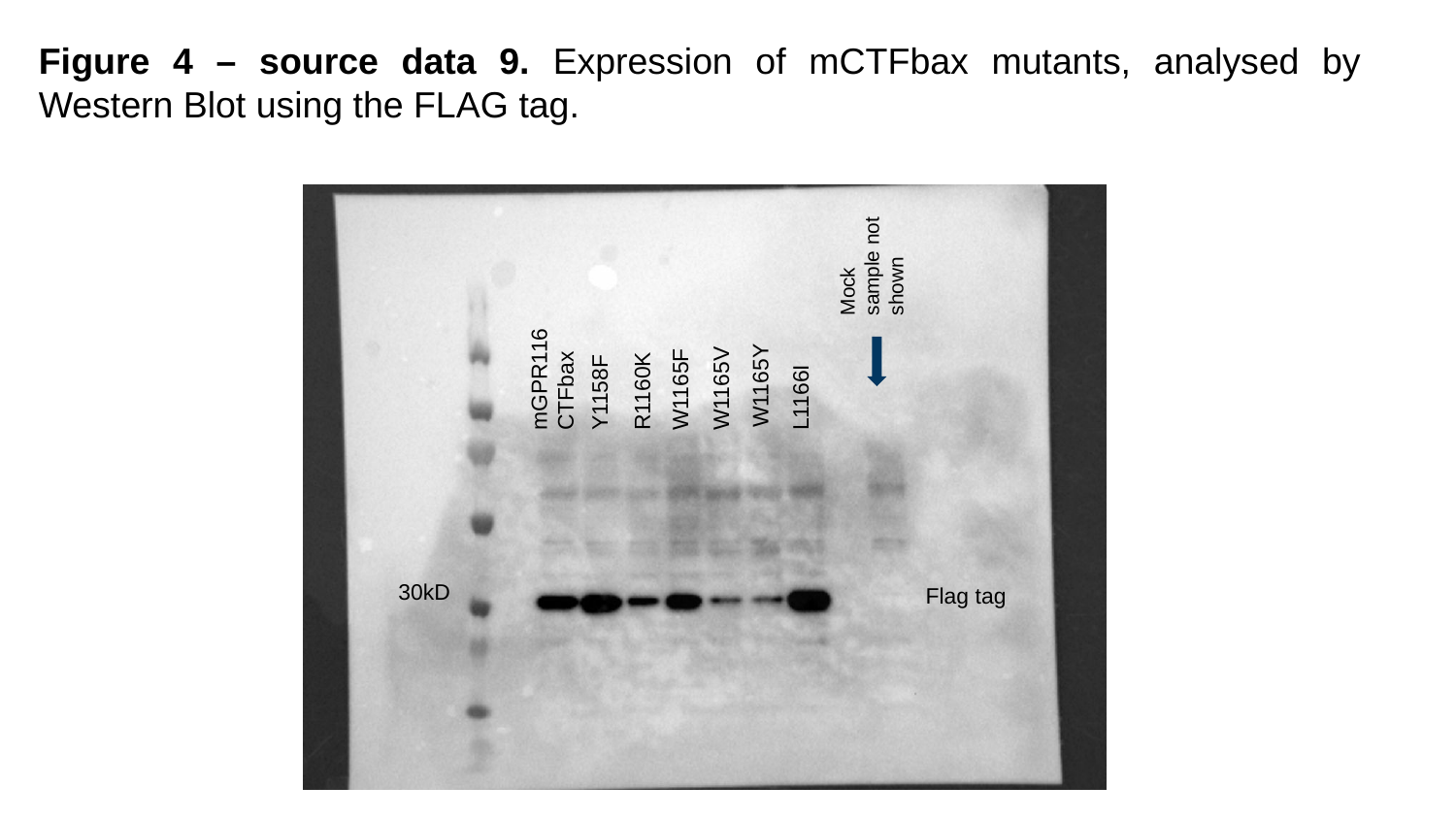

Figure 4 – source data 9. Expression of mCTFbax mutants, analysed by Western Blot using the FLAG tag.
Mock sample not shown
mGPR116 CTFbax
W1165F
W1165Y
R1160K
Y1158F
W1165V
L1166I
30kD
Flag tag

## Slide 2
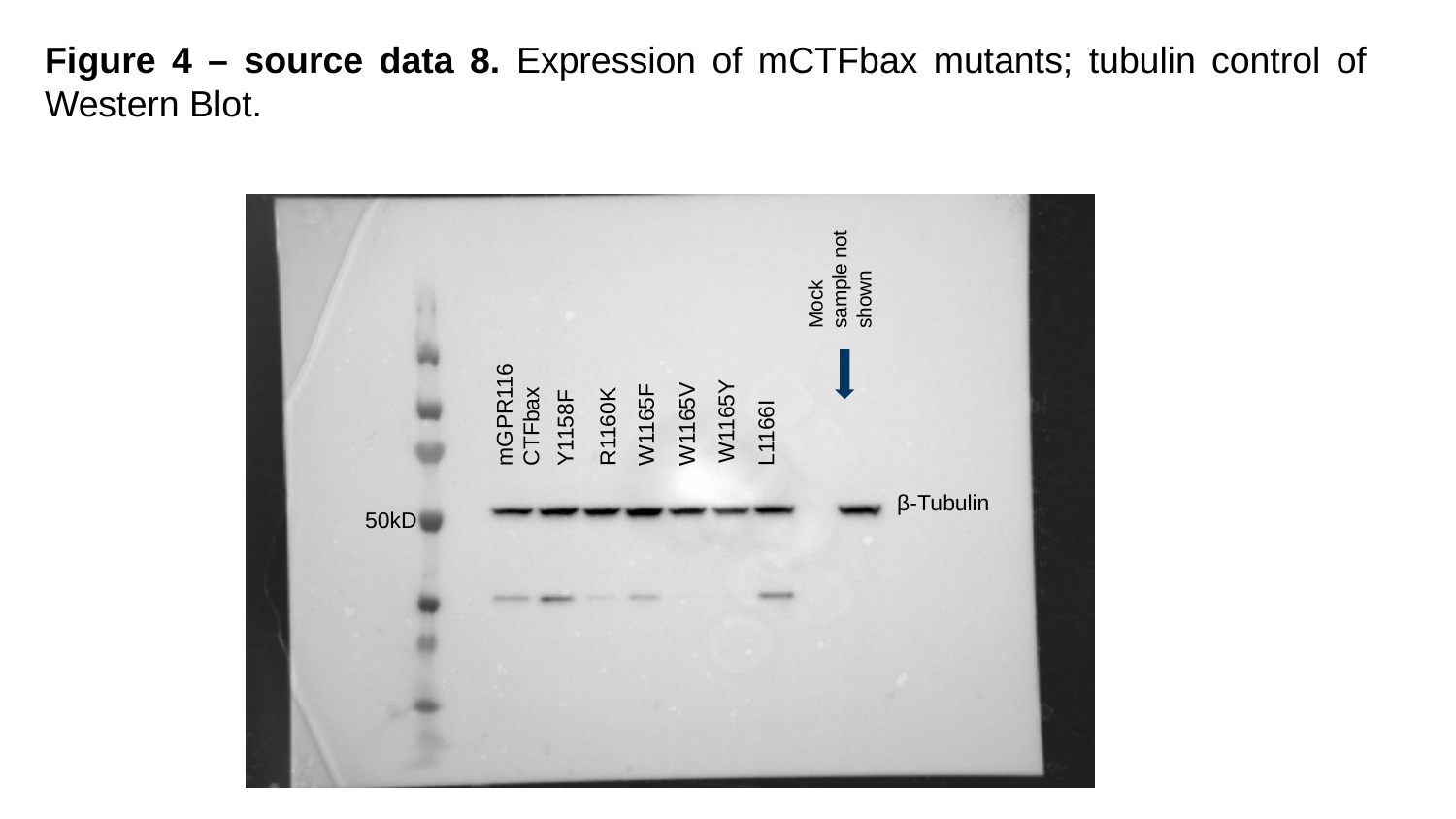

Figure 4 – source data 8. Expression of mCTFbax mutants; tubulin control of Western Blot.
Mock sample not shown
mGPR116 CTFbax
W1165F
W1165Y
R1160K
Y1158F
W1165V
L1166I
β-Tubulin
50kD
