# Supplementary material for: Regulation of pulmonary surfactant by the adhesion GPCR GPR116/ADGRF5 requires a tethered agonist-mediated activation mechanism
Source: eLife. 2022 Sep 8;11:e69061. doi: 10.7554/eLife.69061 (PMC9489211; doi:10.7554/eLife.69061)
Supplement: Figure 5—source data 2. [file elife-69061-fig5-data2.pptx]

## Slide 1
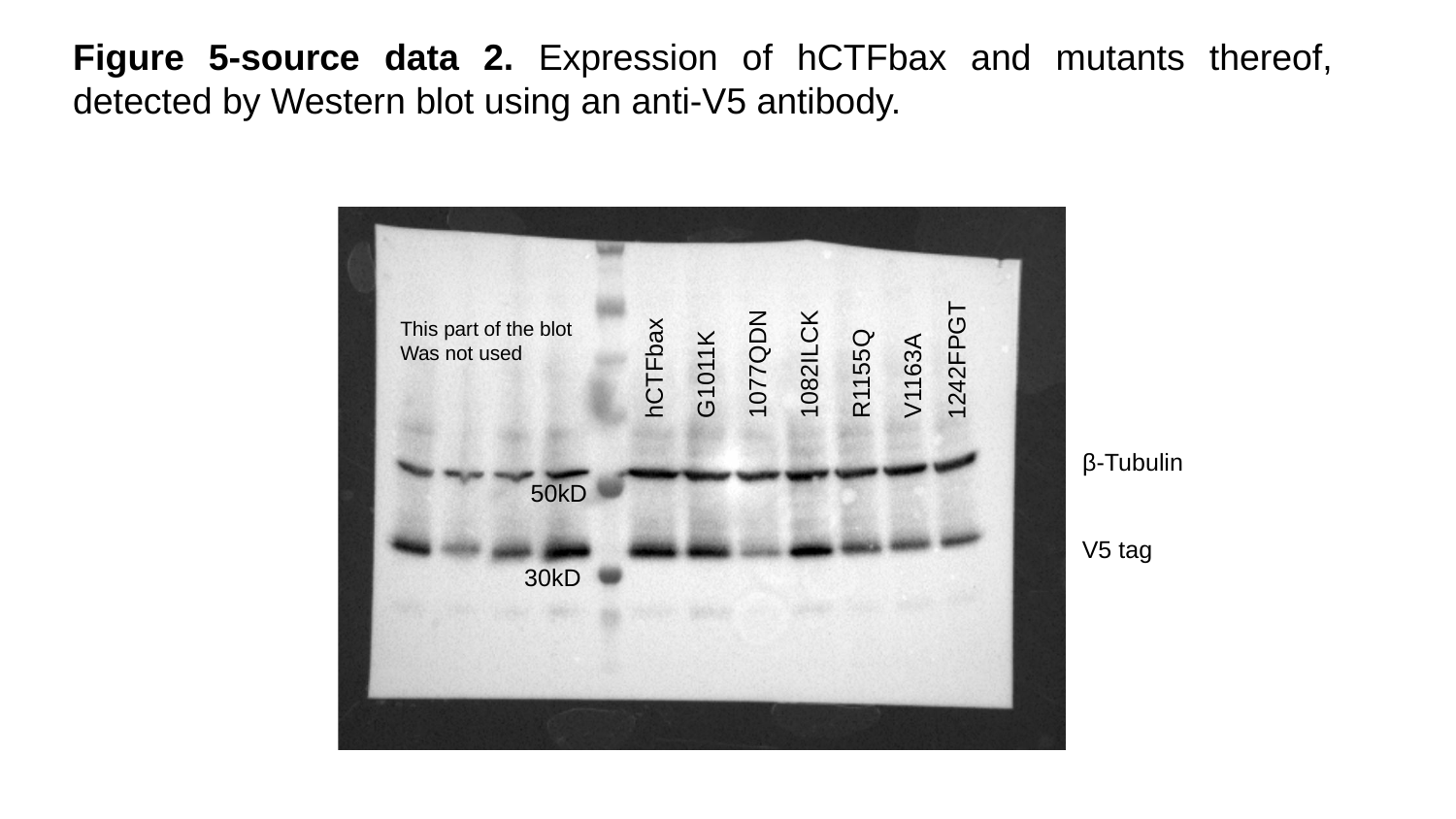

Figure 5-source data 2. Expression of hCTFbax and mutants thereof, detected by Western blot using an anti-V5 antibody.
This part of the blot
Was not used
hCTFbax
G1011K
1077QDN
1082ILCK
R1155Q
V1163A
1242FPGT
β-Tubulin
50kD
V5 tag
30kD
