# Supplementary material for: Regulation of pulmonary surfactant by the adhesion GPCR GPR116/ADGRF5 requires a tethered agonist-mediated activation mechanism
Source: eLife. 2022 Sep 8;11:e69061. doi: 10.7554/eLife.69061 (PMC9489211; doi:10.7554/eLife.69061)
Supplement: Figure 6—source data 2. [file elife-69061-fig6-data2.pptx]

## Slide 1
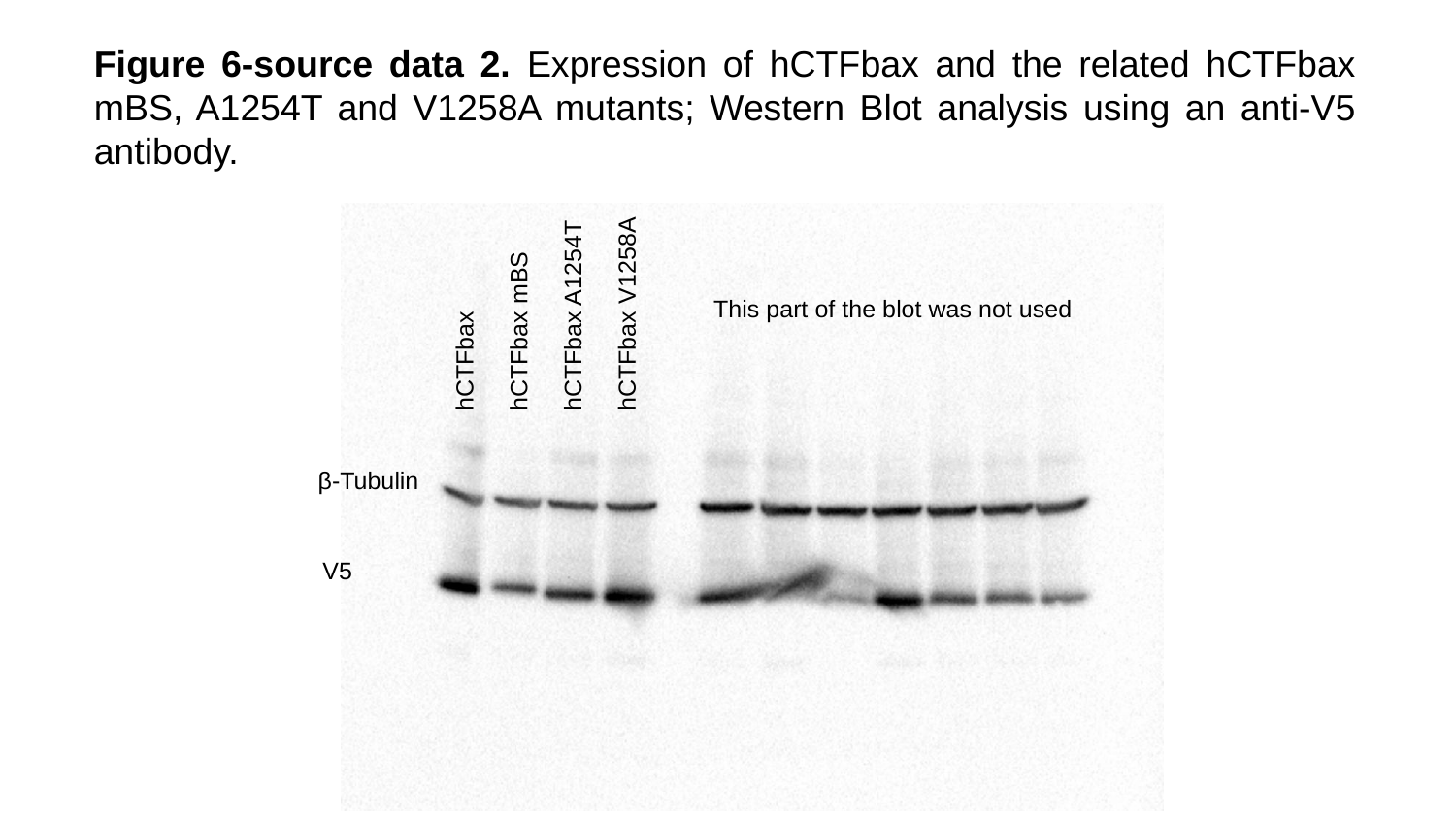

Figure 6-source data 2. Expression of hCTFbax and the related hCTFbax mBS, A1254T and V1258A mutants; Western Blot analysis using an anti-V5 antibody.
hCTFbax V1258A
hCTFbax A1254T
This part of the blot was not used
hCTFbax mBS
hCTFbax
β-Tubulin
V5
