# Supplementary material for: Regulation of pulmonary surfactant by the adhesion GPCR GPR116/ADGRF5 requires a tethered agonist-mediated activation mechanism
Source: eLife. 2022 Sep 8;11:e69061. doi: 10.7554/eLife.69061 (PMC9489211; doi:10.7554/eLife.69061)
Supplement: Supplementary file 1. — Conserved residues mentioned in the text are highlighted in red and bold. Asterisks indicate aGPCRs for which GPS cleavage has not been shown to date. [file elife-69061-supp1.docx]

**Appendix Table 1. Alignment of the human aGPCR tethered agonist and ECL2 sequences.**

Conserved residues mentioned in the text are highlighted in red and bold. Asterisks indicate aGPCRs for which GPS cleavage has not be shown to date.

| **Gene symbol** | **N-terminal part of the CTF (tethered agonist, from GPS cleavage until TM1)** | **ECL2** | **Uniprot** |
| --- | --- | --- | --- |
| ADGRA1* |  | RNYGTEDEDTAY**CW**MAWEPS | **Q86SQ6** |
| ADGRA2* | GNVA**VLM**ELSAFPREVGGAGAGLHP | HNYRDHSPY**CWL**VWRPS | **Q96PE1** |
| ADGRA3* | SNYA**VLM**DLTGSELYTQAASLLHP | KNYGSRPNAPY**CW**MAWEPSLGA | **Q8IWK6** |
| ADGRB1 | ST**F**A**IL**AQLSADANMEKATLPS | AKG**Y**STMNY**CWL**SLEGGLLY | **O14514** |
| ADGRB2 | ST**F**A**VL**AQPPKDLTLELAGSPSVPL | RTKG**Y**GTSSY**CWL**SLEGGLLY | **O60241** |
| ADGRB3 | ST**F**A**IL**AQQPREIIMESSGTPSVT | RTKG**Y**GTDHY**CWL**SLEGGLLY | **O60242** |
| ADGRC1* | AS**F**A**VLM**DISRRENGEVLPLK | PQG**Y**GNPDF**CWL**SLQDTL | **Q9NYQ6** |
| ADGRC2 | TS**F**A**VLM**DVSRRENGEILPLKTLT | PEG**Y**GNPDF**CWL**SIYDTL | **Q9HCU4** |
| ADGRC3* | GT**F**G**VLM**DASPRERLEGDLELLA | PEG**Y**GNPDF**CWI**SVHEP | **Q9NYQ7** |
| ADGRD1 | TN**F**A**ILM**QVVPLELARGHQVALSSIS | DS**Y**GTSNN**CWL**SLASG | **Q6QNK2** |
| ADGRD2* | TS**F**A**IL**LQIYEVQRGPEEESLLRTLS | PHD**Y**VAPGH**CWL**NVHTN | **Q7Z7M1** |
| ADGRE1* | ANLA**V**I**M**ASGELTMD | QPQG**Y**GMHNR**CWL**NTE | **Q14246** |
| ADGRE2 | SS**F**A**VLM**AHYDVQEEDPVLTVIT | RPHL**Y**GTPSR**CWL**QPEKG | **Q9UHX3** |
| ADGRE3 | SS**F**A**VLM**ALTSQEEDPVLT | WPHL**Y**GTADR**CWL**HLDQGFMWSF | **Q9BY15** |
| ADGRE4 | SS**F**A**VL**VALAPKEDPVLT | PQNYGTFT**CWL**KLDKG | **Q86SQ3** |
| ADGRE5 | SS**F**A**ILM**AHYDVEDWKLTLITR | SKG**Y**GRPRY**CWL**DFEQG | **P48960** |
| ADGRF1 | TS**F**S**ILM**SPFVPSTIFPVVKWITY | TQPSNT**Y**K**R**KDV**CWL**NWSNGSKPL | **Q5T601** |
| ADGRF2* | TS**F**S**ILM**SPHILESLILTYITY | VAATEPGKG**Y**L**R**PEI**CWL**NWDMTKALLA | **Q8IZF7** |
| ADGRF3 | TA**F**S**VLM**SPHTVPEEPALALLTQ | GLYLPQGQ**Y**L**R**EGE**CWL**DGKGGALYT | **Q8IZF5** |
| ADGRF4* | MS**F**S**ILM**SSKSMTDKVLDYITC | TEPEKG**Y**M**R**PEA**CWL**NWDNTKALLA | **Q8IZF3** |
| ADGRF5 | TS**F**S**ILM**SPDSPDPSS | TQPREV**Y**T**R**KNV**CWL**NWEDTKALL | **Q8IZF2** |
| ADGRG1 | TY**F**A**VLM**VSSVEVDAVHKHY | DNYGPIILAVHRTPEGVIYPSM**CWI**RDSLVSYITNLG | **Q9Y653** |
| ADGRG2 | TS**F**G**VL**LDLSRTSVLPAQMMA | DNYGLGSYGKFPNGSPDDF**CWI**NNNAVFYIT | **Q8IZP9** |
| ADGRG3 | TF**F**AL**L**LRPTLDQSTVHILTRIS | GTGSANSYGLYTIRDRENRTSLEL**CW**FREGTTMYALYIT | **Q86Y34** |
| ADGRG4 | TH**F**G**VLM**DLSRSTVDSVNEQ | SVKKDLYGTLSPTTPF**CWI**KDDS | **Q8IZF6** |
| ADGRG5* | TY**F**A**VLM**QLSPALVPAELLAPLTY | SVKSSVYGPCTIPVFDSWENGTGFQNMSI**CW**VRSPVVHS | **Q8IZF4** |
| ADGRG6 | TH**F**G**VLM**DLPRSASQLDARNTK | SRNNNEVYGKESYGKEKGDEF**CWI**QDPVIFYVT | **Q86SQ4** |
| ADGRG7* | TN**F**A**VLM**TFKKDYQYPKSLDILS | GVIYSQNGNNPQWELD**Y**RQEKI**CWL**AIPEPNGVIKSP | **Q96K78** |
| ADGRL1 | TN**F**A**VLM**AHREIYQGRINEL | YRS**Y**GTEKA**CWL**RVDNY | **O94910** |
| ADGRL2 | TN**F**A**ILM**AHREIAYKDGVHELLLTVIT | KS**Y**GTEKA**CWL**HVDNYF | **O95490** |
| ADGRL3 | TN**F**A**VLM**AHVEVKHSDAVHDLLLDV | DYRS**Y**GTDKV**CWL**RLDTYF | **Q9HAR2** |
| ADGRL4 | TH**F**A**ILM**SSGPSIGIKDYNILTRITQ | RY**Y**GTTKV**CWL**STENNFIWS | **Q9HBW9** |
| ADGRV1 | SVYA**V**YARTDNLSSYNEA | LKGIYHQSMSQIYGLIHGDL**C**F**I**PNVYA | **Q8WXG9** |
