# Supplementary material for: Regulation of pulmonary surfactant by the adhesion GPCR GPR116/ADGRF5 requires a tethered agonist-mediated activation mechanism
Source: eLife. 2022 Sep 8;11:e69061. doi: 10.7554/eLife.69061 (PMC9489211; doi:10.7554/eLife.69061)
Supplement: Supplementary file 2. — EC50s, measured in the calcium transients assay, are given in µM. Number of data points, agonistic peptide used, mean ± SD, and statistical significance are detailed. n.d., not determined; ns, not significant. [file elife-69061-supp2.docx]

**Appendix Table2. Potency of the various constructs activated by GAPs.**

EC50s, measured in the calcium transients assay, are given in uM. Number of data points, agonistic peptide used and statistical significance are detailed. n.d. not determined, ns not significant.

|  | **Construct** | **GAP** | **Data nb** | **EC50 (uM)** |  | **Statistics** | |  |  |  |  |
| --- | --- | --- | --- | --- | --- | --- | --- | --- | --- | --- | --- |
| **Fig4B** | mCTFbax | GAP14 | n=3 | 152 +/- 65 |  |  | transient |  |  |  |  |
|  | mutants | GAP14 | n=3 | n.d. |  | p<0.0001 | mCTFbax vs. mutants |  |  |  |  |
|  |  |  |  |  |  |  |  |  |  |  |  |
| **Fig4H** | mFL 1D8 | GAP14 | n=3 | 124 +/- 49 |  |  |  |  |  |  |  |
|  | mutants | GAP14 | n=3 | n.d. |  | p<0.0001 | mFL vs. mutants |  |  |  |  |
|  |  |  |  |  |  |  |  |  |  |  |  |
| **Fig4I** | mCTFbax | GAP14 | n=3 | 74 +/- 28 |  | TTEST |  |  |  |  |  |
|  | Y1158F | GAP14 | n=3 | 226 +/- 46 |  | 0.0179 |  |  |  |  |  |
|  |  |  |  |  |  |  |  |  |  |  |  |
|  | mCTFbax | GAP14 | n=3 | 92 +/- 28 |  | ns |  |  |  |  |  |
|  | T1240S | GAP14 | n=3 | 69 +/- 16 |  |  |  |  |  |  |  |
|  |  |  |  |  |  |  |  |  |  |  |  |
| **Fig5B** | hCTFbax | GAP14 | n=3 | 345 +/- 28uM |  | TTEST |  |  |  |  |  |
|  | mCTFbax | GAP14 | n=3 | 175 +/- 38 M |  | 0.0035 |  |  |  |  |  |
|  |  |  |  |  |  |  |  |  |  |  |  |
| **Fig5E** | hCTFbax | GAP14 | n=8 | 971 +/- 609 |  |  |  |  |  |  |  |
|  | mCTFbax | GAP14 | n=5 | 196 +/- 46 |  | p<0.05 | hCTFbax vs. mCTF |  |  |  |  |
|  | 1077QDN | GAP14 | n=5 | 381+/- 86 |  | ns | hCTFbax vs. 1077QDN |  |  |  |  |
|  | 1082ILCK | GAP14 | n=6 | 862 +/- 639 |  | ns | hCTFbax vs. 1082ILCK |  |  |  |  |
|  |  |  |  |  |  |  |  |  |  |  |  |
|  | hCTFbax | GAP14 | n=5 | 1227 +/- 664 |  | TTEST |  |  |  |  |  |
|  | mCTFbax | GAP14 | n=5 | 229 +/- 106 |  | 0.0106 |  |  |  |  |  |
|  | R1155Q | GAP14 |  | n.d. |  |  |  |  |  |  |  |
|  | 1242FPGT | GAP14 |  | n.d. |  |  |  |  |  |  |  |
|  |  |  |  |  |  |  |  |  |  |  |  |
|  | hCTFbax | GAP14 | n=5 | 1261 +/- 604 |  |  |  |  |  |  |  |
|  | mCTFbax | GAP14 | n=6 | 186 +/- 74 |  | p<0.01 | hCTFbax vs. mCTF |  |  |  |  |
|  | G1011K | GAP14 | n=5 | 374 +/- 54 |  | p<0.01 | hCTFbax vs. G1011K |  |  |  |  |
|  | V1163A | GAP14 |  | n.d. |  |  |  |  |  |  |  |
|  |  |  |  |  |  |  |  |  |  |  |  |
| **Fig6B** | hCTFbax | GAP14 | n=3 | 435 +/- 8 |  | p<0.001 | hCTFbax vs. mCTFbax |  |  |  |  |
|  | mCTFbax | GAP14 | n=3 | 54 +/- 27 |  | p<0.001 | hCTFbax vs. hCTFbax mBS |  |  |  |  |
|  | hCTFbax mBS | GAP14 | n=3 | 162 +/- 74 |  | p<0.05 | mCTFbax vs. hCTFbax mBS |  |  |  |  |
|  |  |  |  |  |  |  |  |  |  |  |  |
| **Fig6C** | hCTFbax | GAP14 | n=3 | 485 +/- 269 |  | p<0.05 | hCTFbax mBS vs. hCTFbax |  |  |  |  |
|  | hCTFbax mBS | GAP14 | n=3 | 54 +/- 21 |  |  |  |  |  |  |  |
|  | V1258A | GAP14 | n=3 | 77 +/- 14 |  | ns | hCTFbax mBS vs. V1258A |  |  |  |  |
|  | A1254T | GAP14 | n=3 | 161 +/- 128 |  | ns | hCTFbax mBS vs. A1254T |  |  |  |  |
|  |  |  |  |  |  |  |  |  |  |  |  |
| **Fig7A** | mFL 3C | GAP14 | n=4 | 12.5 +/- 3.5 |  | p<0.05 | GAP10 vs. GAP14 |  |  |  |  |
|  | mFL 3C | GAP10 | n=4 | 6.7 +/- 1.9 |  |  |  |  |  |  |  |
|  | mFL 3C | GAP10-Pro1 | n=4 | 5.1 +/- 1.4 |  | ns | GAP10 vs. GAP10-Pro1 |  |  |  |  |
|  |  |  |  |  |  |  |  |  |  |  |  |
|  | hFL A6 | GAP14 | n=3 | 242 +/- 60 |  | p<0.01 | GAP10 vs. GAP14 |  |  |  |  |
|  | hFL A6 | GAP10 | n=3 | 100 +/- 12 |  |  |  |  |  |  |  |
|  | hFL A6 | GAP10-Pro1 | n=3 | >500 |  | p<0.0001 | GAP10 vs. GAP10-Pro1 |  |  |  |  |
|  |  |  |  |  |  |  |  |  |  |  |  |
|  | mCTFbax | GAP14 | n=3 | 247 +/- 113 |  | p<0.01 | GAP10 vs. GAP14 |  |  |  |  |
|  | mCTFbax | GAP10 | n=4 | 65 +/- 19 |  |  |  |  |  |  |  |
|  | mCTFbax | GAP10-Pro1 | n=4 | 22 +/- 7 |  | ns | GAP10 vs. GAP10-Pro1 |  |  |  |  |
|  |  |  |  |  |  |  |  |  |  |  |  |
|  | hCTFbax | GAP14 | n=3 | 515 +/- 163 |  | ns | GAP10 vs. GAP14 |  |  |  |  |
|  | hCTFbax | GAP10 | n=3 | 300 +/- 97 |  |  |  |  |  |  |  |
|  | hCTbax | GAP10-Pro1 | n=3 | n.d. |  | n.d. | GAP10 vs. GAP10-Pro1 |  |  |  |  |
|  |  |  |  |  |  |  |  |  |  |  |  |
| **Fig7B** | hCTFbax | GAP10 | n=4 | 736 +/- 389 |  | p<0.001 | hCTFbax mBS vs. hCTFbax |  | **Fig7B** |  |  |
|  | hCTFbax mBS | GAP10 | n=4 | 48 +/- 9 |  |  |  |  | **stats for maximal efficacy** | uM |  |
|  | hCTFbax A1254T | GAP10 | n=4 | 99 +/- 23 |  | ns | hCTFbax mBS vs. A1254T |  | hCTFbax mBS vs. hCTFbax | 500 | p<0.0001 |
|  | hCTFbax V1258A | GAP10 | n=4 | 71 +/- 25 |  | ns | hCTFbax mBS vs. V1258A |  | hCTFbax mBS vs. hCTF bax A1254T |  | p<0.0001 |
|  |  |  |  |  |  |  |  |  | hCTFbax mBS vs. hCTF bax V1258A |  | p<0.01 |
|  | hCTFbax | GAP10-Pro1 | n=4 | >500 |  | p<0.01 | hCTFbax mBS vs. hCTFbax |  |  |  |  |
|  | hCTFbax mBS | GAP10-Pro1 | n=4 | 32 +/- 13 |  |  |  |  | hCTFbax mBS vs. hCTFbax | 250 | p<0.0001 |
|  | hCTFbax A1254T | GAP10-Pro1 | n=4 | 89 +/- 29 |  | ns | hCTFbax mBS vs. A1254T |  | hCTFbax mBS vs. hCTF bax A1254T |  | p<0.01 |
|  | hCTFbax V1258A | GAP10-Pro1 | n=4 | 67 +/- 22 |  | ns | hCTFbax mBS vs. V1258A |  | hCTFbax mBS vs. hCTF bax V1258A |  | p<0.05 |
|  |  |  |  |  |  |  |  |  |  |  |  |
| **Fig7C** | hCTFbax - GAP10 |  | n=3 | 268 +/- 64 |  |  |  |  | hCTFbax mBS vs. hCTFbax | 125 | p<0.0001 |
|  | hCTFbax - GAP10-Pro1 | | n=3 | n.d. |  | TTEST |  |  | hCTFbax mBS vs. hCTF bax A1254T |  | p<0.0001 |
|  | hCTFbax G1011K - GAP10 | | n=3 | 137 +/- 49 |  | p<0.05 | hCTFbax vs. G1011K |  | hCTFbax mBS vs. hCTF bax V1258A |  | p<0.0001 |
|  | hCTFbax G1011K - GAP10-Pro1 | | n=3 | n.d. |  |  |  |  |  |  |  |
|  |  |  |  |  |  |  |  |  |  |  |  |
| **Fig S3** | hFL A6 | GAP10 | n=3 | 101+/-30 |  |  |  |  |  |  |  |
|  | hFL A6 | Ala mutants | n=3 | n.d. |  |  |  |  |  |  |  |
|  |  |  |  |  |  |  |  |  |  |  |  |
| **Fig S6A** | hFL A6 | GAP14 | n=3 | 138+/-28 |  |  |  |  |  |  |  |
|  | mutants | GAP14 | n=3 | n.d. |  |  |  |  |  |  |  |
